# Redo mitral surgery after coronary artery bypass grafts under hyperkalemic hypothermia using thoracotomy and axillary artery cannulation in a patient with functioning bilateral internal thoracic arteries and atheromatous aorta

**DOI:** 10.1186/s13019-023-02209-1

**Published:** 2023-04-17

**Authors:** Ryo Suzuki, Masafumi Akita, Takaki Itohara, Takuya Komatsu

**Affiliations:** Department of Cardiovascular Surgery, Shinmatsudo Central General Hospital, 1-380 Shinmatsudo, Matsudo, Chiba 270-0034 Japan

**Keywords:** Hypothermic hyperkalemic cardiac arrest, Internal thoracic artery, Redo mitral valve surgery, Hypothermic fibrillatory arrest

## Abstract

**Background:**

Redo mitral valve surgery using resternotomy after coronary artery bypass grafting (CABG) is challenging as previous CABG with patent internal thoracic artery (ITA) poses a risk of injury due to dense adhesion. It is paramount to have alternative method to minimize this risk.

**Case presentation:**

: We report a case of redo mitral and tricuspid valve repair via right thoracotomy under hypothermia and systemic potassium administration with axillary artery cannulation in a patient after CABG with patent bilateral ITA grafts crossing over the sternum. Herein, critical dissection around the aorta and functioning ITA grafts was avoided by performing the procedure under systemic hypothermia via thoracotomy. Furthermore, considering the presence of atheroma in the aorta, the axillary artery was used as a perfusion route to prevent stroke events. Postoperative course was uneventful and echocardiography demonstrated preserved cardiac function.

**Conclusion:**

Performing axillary artery cannulation and right thoracotomy under hypothermic cardiac arrest with systemic hyperkalemia without clamping the patent bilateral ITAs and aorta allowed us to perform redo mitral valve surgery after CABG without major postoperative cardiac or cerebral complications.

## Background

Mitral valve surgery in patients with a history of coronary artery bypass grafting (CABG) can be challenging for cardiac surgeons. As patent internal thoracic artery (ITA) graft injury during reoperation via resternotomy in the setting of dense adhesion is often fatal, it is essential to establish an alternative protocol to minimize potential injury to ITA grafts. Previous studies have reported that redo mitral valve surgery can be safely performed via right thoracotomy without clamping ITAs under hypothermic cardiac arrest after initial CABG with patent ITA grafts [1]. Here, we report a case of redo mitral valve repair via right thoracotomy under hypothermia and hyperkalemia without clamping the patent bilateral ITAs after previous CABG using axillary artery cannulation as an alternative.

## Case presentation

**Fig. 1:**
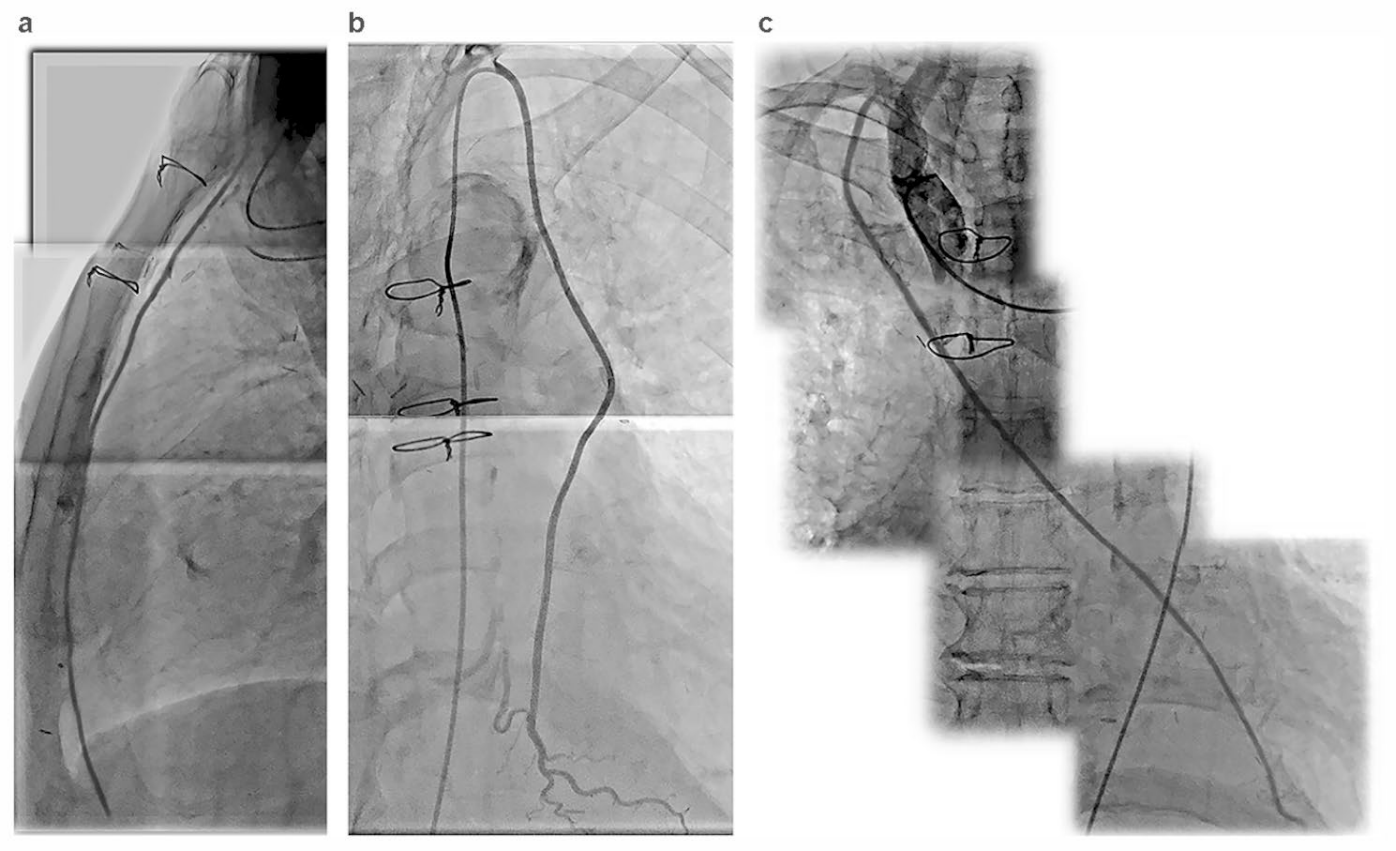
(a) Angiographic view of the patent internal thoracic artery (ITA) running close to the sternum and aorta. (b) Patent left ITA. (c) Right ITA over the sternum

A 77-year-old woman who had undergone CABG with bilateral ITAs 6 years ago, with right ITA to left anterior descending artery and left ITA to obtuse marginal artery, presented to our hospital with symptoms of heart failure and was diagnosed with severe mitral and tricuspid regurgitation (Fig. 1a–c). Echocardiography revealed a dilated mitral annulus and central severe mitral regurgitation jet due to atrial fibrillation. Structural interventions were recommended. However, surgical mitral and tricuspid valve repair was performed for mitral and tricuspid regurgitation because addressing them simultaneously may improve the clinical outcome.

**Fig. 2:**
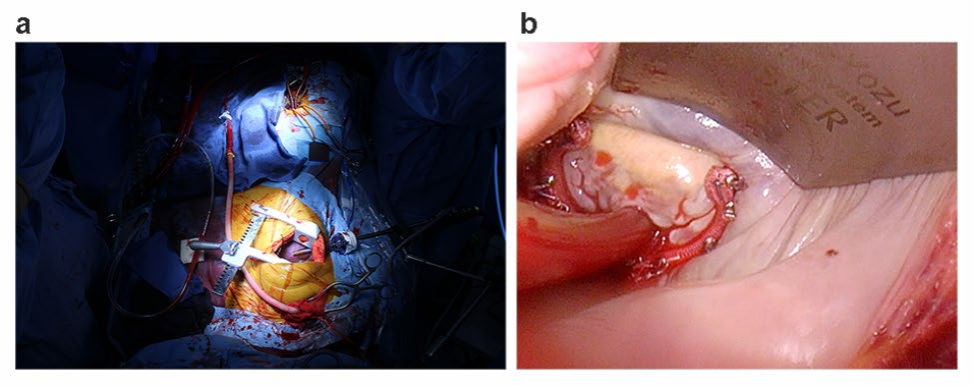
(a) To visualize the valves, cardiopulmonary bypass was performed via axillary artery cannulation and right thoracotomy (b) Endoscopic view of the mitral valve after ring annuloplasty

Thoracotomy rather than resternotomy was performed because the patent ITA graft crossed over the sternum (Fig. 1a, c). An 8-mm Dacron tube graft was connected to the right axillary artery through which cardiopulmonary bypass was established, thereby preventing embolism due to atheroma burden in the ascending aorta and reducing the risk of ITA injury as the graft ran close to the ascending aorta (Fig. 1a). Accordingly, aorta was not clamped. The femoral vein and superior vena cava were cannulated for venous drainage. Her temperature was maintained at 24 °C, and systemic potassium was administered at a concentration of 40 mEq/L. Further, potassium was periodically administered to maintain a concentration of > 7 mEq/L. Total given potassium was 180 mEq and was added in the reservoir as total of 180ml solution. Thus, cardiac arrest was achieved, the fourth right intercostal space was entered to access to the valves. And a camera port was placed in the fifth intercostal space along the mid axillary line (Fig. 2a). The venting cannula was placed through the right upper pulmonary vein to the left atrium and another venting cannula was put in the left ventricular through the thoracotomy across the mitral valve. The carbon dioxide insufflation was performed to prevent air embolism. Suction at the level of 20-60mmHg was applied to the reservoir for better venous drainage and blood-less operating filed. The dilated mitral and tricuspid annulus was fixed using an annuloplasty ring (Fig. 2b). She was returned to normothermic conditions, and a spontaneous heartbeat was restored. The duration of cardiac arrest was 135 min, whereas that of cardiopulmonary bypass was 218 min. Postoperative echocardiography revealed trivial mitral and tricuspid regurgitation and preserved cardiac function with an ejection fraction of 64%.

## Discussion and conclusions

The optimal strategy for myocardial protection during reoperative cardiac surgery after initial CABG with patent ITA graft remains controversial. Some researchers believe that ITA dissection and clamping should be avoided as the risk of ITA injury outweighs the benefits of identifying and controlling the ITA graft [2]; conversely, others believe that leaving the ITA open during cardiopulmonary bypass causes biventricular hypokinesis due to cardioplegia washout by the patent ITA graft, resulting in inadequate myocardial protection. A previous study has suggested that reoperative cardiac surgery can be safely performed in patients with previous CABG and patent ITA using systemic hyperkalemia and hypothermic arrest without clamping the ITA graft [3]. In our case, the bilateral ITAs were functional and left unclamped; however, the maximum level of postoperative creatine kinase-MB was 100 U/L with reasonable myocardial protection. When graft perfusion washes out cardioplegia and restores the electromechanical activity, the myocardial oxygen consumption increases, thereby impairing myocardial protection [4]. A study reported the use of hypothermic fibrillatory arrest (HFA) has been used in such cases; however, it may lead to heterogenous myocardial protection in the setting of patent graft flow to the heart. In addition, HFA is associated with compromised subendocardial perfusion [5]. During ventricular fibrillation, the myocardium is still continuously contracting, thereby missing the complete flaccid diastolic phase. Blood flow to the subendocardium occurs during diastole. Therefore, the compressive force exerted on the subendocardial muscle by fibrillation restricts the blood flow and oxygen delivery to the subendocardium during ventricular fibrillation [5]. Whereas, systemic hyperkalemia provides complete electromechanical diastolic arrest with uniform myocardial protection throughout the heart as opposed to fibrillatory arrest without the fear of cardioplegia washout [4]. In this case, the procedure was rather complex, with mitral and tricuspid surgeries requiring a long cardiac arrest time. Therefore, systemic hyperkalemic arrest might be considered more suitable and safe option.

The right minithoracotomy approach provides an excellent operative view of the mitral valve without requiring the dissection of the adhesion and ITA grafts after CABG with median sternotomy. Evidence also shows that redo mitral surgery via the right thoracotomy reduces mortality as well as shorter hospital stay compared with remedian sternotomy [6].

Owing to the antegrade perfusion of the brain, axillary artery cannulation is more effective than femoral artery cannulation in protecting the brain and preventing stroke events during aortic surgeries requiring hypothermic circulatory arrest [7]. Also, Shiiya and colleagues demonstrated that the isolation selective cerebral perfusion technique was effective in preventing stroke events during aortic arch surgery, in which axillary artery perfusion was performed to prevent the entry of emboli into the brain circulation system as a stroke prevention measure [8]. Considering the presence of atheroma in the ascending aorta, we perfused through the axillary artery for cardiopulmonary bypass without major postoperative cerebral complications.

In this case, the combination of axillary artery cannulation and right thoracotomy under hypothermic cardiac arrest with systemic hyperkalemia without clamping the patent bilateral ITAs and aorta enabled safe surgery without major postoperative cardiac or cerebral complications in a patient with atheromatous aorta and previous CABG with patent bilateral ITAs.

## Data Availability

Data associated with this article will be shared on reasonable request to the corresponding author.

## List of abbreviations

CABG: Coronary artery bypass grafting
ITA: Internal thoracic artery
HFA: Hypothermic fibrillatory arrest
